# The challenges to detect, quantify, and characterize viral reservoirs in the current antiretroviral era

**DOI:** 10.1515/nipt-2024-0017

**Published:** 2024-12-05

**Authors:** Hector Gutierrez, Eliseo A. Eugenin

**Affiliations:** Department of Neurobiology, The University of Texas Medical Branch (UTMB), Galveston, TX, USA

**Keywords:** AIDS, HIV, reservoirs, cure

## Abstract

A major barrier to cure HIV is the early generation of viral reservoirs in tissues. These viral reservoirs can contain intact or defective proviruses, but both generates low levels of viral proteins contribute to chronic bystander damage even in the ART era. Most viral reservoir detection techniques are limited to blood-based, reactivation, and sequencing assays that lack spatial properties to examine the contribution of the host’s microenvironment to latency and cure efforts. Currently, little is known about the contribution of the microenvironment to viral reservoir survival, residual viral expression, and associated inflammation. Only a few spatiotemporal techniques are available, and fewer integrate spatial genomics, transcriptomics, and proteomics into the analysis of the viral reservoir microenvironment-all essential components to cure HIV. During the development of these spatial techniques, many considerations need to be included in the analysis to avoid misinterpretation. This manuscript tries to clarify some critical concepts in viral reservoir detection by spatial techniques and the upcoming opportunities for cure efforts.

## General

**Figure 1:**
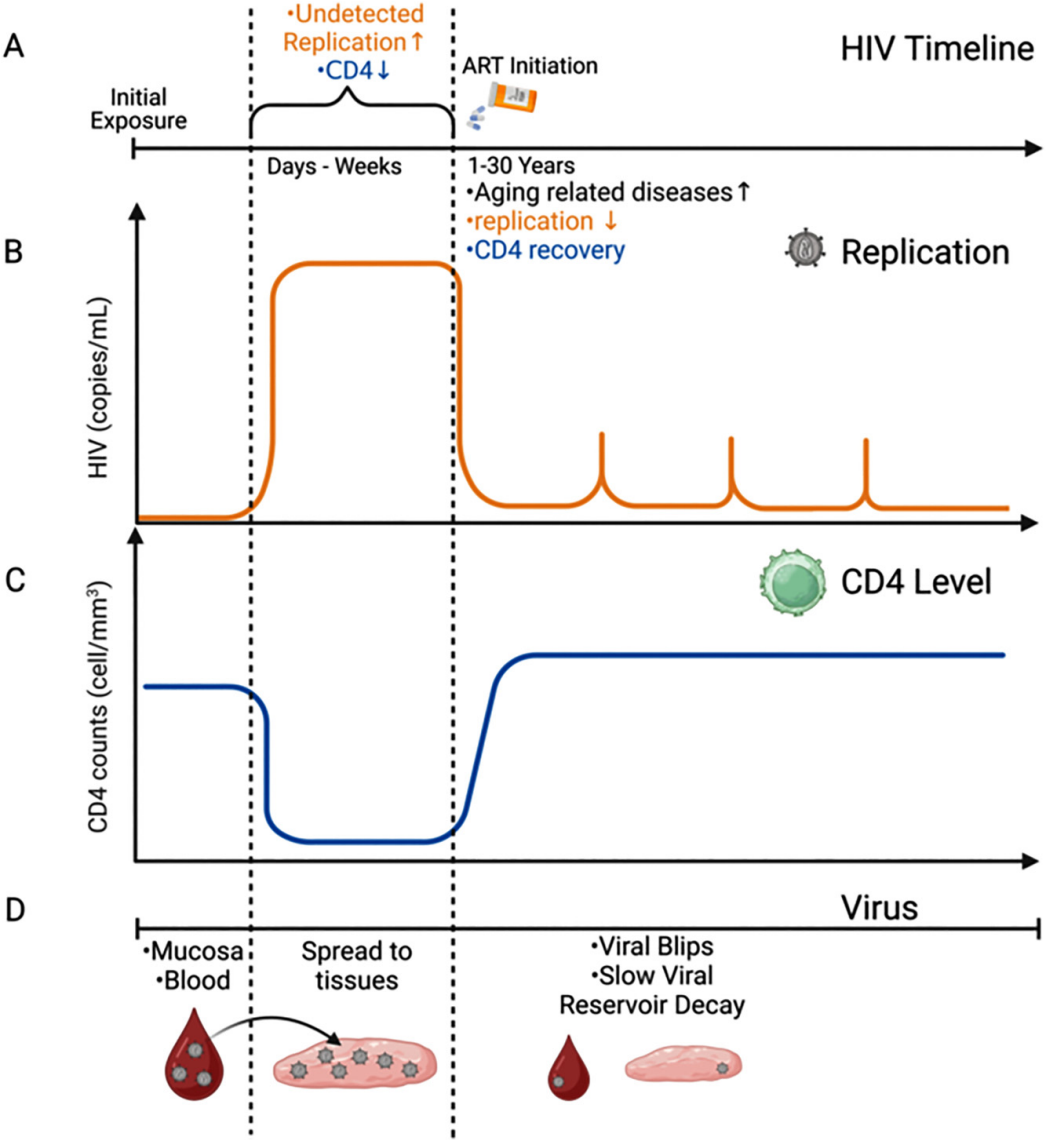
A graphical representation of the time course of HIV infection (A) and replication in the presence of ART (B) and the relative consequences of CD4 counts (C). (D) Represents the initial infection, tissue spread (viral reservoir stability), and long-term viral decay (Figure created in Biorender).

HIV/AIDS represents a significant global public health concern, with an estimated 38 million infected individuals worldwide and 1.2 million in the US. Despite the substantial success of antiretroviral therapies (ART) in controlling systemic HIV replication, these therapies are not a cure. The main obstacle to cure HIV-infected individuals is the early colonization of most tissues with HIV-infected cells that later become viral reservoirs [1, 2] (see Figure 1).

Currently, in virologically suppressed HIV-infected individuals, several types of viral reservoirs in blood products have been identified, including in CD4^+^ T lymphocytes [3, 4], but also in tissues including the brain [5, 6], urethra [7], gut [8], and liver [9]. Data from patients indicates that viral rebound upon ART interruption is mediated by the contribution of multiple anatomical sources, including the brain, suggesting that viral reservoirs have numerous identities and hide in various tissues [10] (Figure 1).

Both of Siliciano’s groups are leaders in detecting and quantifying viral reservoirs 11], [12], [13. Their calculations indicate that viral reservoirs within the blood after long-term ART are rare 14], [15], [16. Analysis after ∼7.1 years after ART initiation by Intact Proviral DNA Assay (IPDA) as total *integrated* DNA by qPCR indicates a mean of 269–208 copies per million CD4^+^ T cells with hypermutated 3′ defective and 5′ defective proviruses; in contrast, only 64.6 copies per million were identified for full-length intact proviruses, with a half life of 7.1 years [14]. Analysis by quantitative viral outgrowth assay (QVOA) indicates that the half life of the viral reservoir is 3.6 years [14, 15, 17, 18]. These discrepancies may be related to different latency stages, reactivation requirements or method of analysis; in agreement, intact proviruses accumulate in non-genic chromosomal positions that could explain the deeper latency of intact viruses [19]. However, as will be discussed below, both cell types, intact and defective, produce residual expression of viral proteins, inducing local inflammation. Overall, these data show that the decay of the viral reservoir pool in the circulation is extremely slow and not achievable in a lifetime (t_1/2_ =3.6 years). By estimation, a starting number of one million cells harboring the viral reservoir would take 73 years of therapy to eradicate it, and unfortunately, ART poorly controls these numbers [20, 21]. Thus, eradication is unlikely possible through simple long-term ART alone, and new treatments or strategies are required to reach a cure.

Another source of variability that requires consideration is the decay of the genome-intact viruses that differ from the genome-defective pro-viruses upon ART [15, 22, 23]. These data suggest that viral reservoirs could adapt to ART, and their microenvironment probably further contributing to their variability in nature, and survival mechanisms [24]. Likely, decay of the viral reservoir pool is cell type, tissue, microenvironment, and ART-specific, but better technologies are required to evaluate these observations. This then begs the question: why do these viral reservoirs persist for a long time? Six hypotheses have been described. First, a suboptimal concentration of ART in different tissues and within different areas of the same tissue. Second, residual viral replication and lower cell activation that help to protect the HIV-infected cells from anti-HIV immune responses such as cytotoxic CD8^+^ T cells. Third, for macrophages and astrocytes, several viral proteins prevent immune recognition and apoptosis. Fourth, every viral reservoir is different because of multiple differentiation stages that are present, including cell type, tissue residence (brain, gut, spleen, lymph nodes, and others), and length. Fifth, there is long-term stability due to cell renewal or proliferation. Lastly, the microenvironment associated with these viral reservoirs may have an impact as well. Little is known about many of these hypotheses, and defining these mechanisms could generate new therapeutics to cure HIV.

## Methodologies to detect viral reservoirs

Clinical assays to detect systemic viral replication do not determine the viral reservoir size [13]. However, there are more than 100 clinical trials examining cure strategies, including new techniques to measure viral reservoir pools to demonstrate eradication (https://www.treatmentactiongroup.org/cure/trials/). However, a significant issue in all current methods to detect viral reservoirs are related to sensitivity, resolution, and specificity, as well as many rely on specific amplification protocols not representative of viral reactivation present in humans. For example, latency reverting agents (LRA) only reactivate one pool of viral reservoir [13, 25]. Currently, most viral reservoir methodologies focus on detecting one viral component, including viral DNA, mRNA, episomal DNA, viral proteins, or viral secretion [1, 13, 26]. Also techniques to detect viral reservoirs rely on whole genome sequencing or reactivation protocols, and all lack the spatial resolution to detect them and their associated microenviroment. The gold standard for viral detection is based on the amplification of the proviral genome by PCR-based assays in baseline conditions or after reactivation with specific agents. The best-known assay to detect viral reservoirs is QVOA [27, 28]. However, the major limitations of QVOA are that it is expensive, laborious, requires a high cell number, is blood-based, is a selecting reactivation agent, and there is no determined non-competent viruses providing the minimum estimate of the viral reservoir size. IPDA and others thecniques provide an extremely useful tool to identify intact and non-intact proviruses. All tests are based on next-generation sequencing and subsequent analysis [27, 28]. Other techniques are based on single-cell sequencing, including single-cell sorting, proteogenomic profiling (FIND-seq or PheP-seq), and single-cell transcriptomics/epigenetics [29]. Further, proviral sequences are characterized by single genome near full-length proviral sequencing (FLIP-seq/FLIPS assays), Q4PCR, or IPDA [20, 30], [31], [32. In HIV RNA detection, we believe that Tat-/Rev- Induced limiting dilution assay is one of the best RNA PCR detection techniques [27, 28]. But all these tests determine only 1 component of the viral replication cycle-an insufficient determination to study the stage of latently infected cells in cells, blood, and tissues.

Also, we have to denote that it has become evident that non-fully replicating or defective viruses are still capable of transcribing some viral RNA and producing viral proteins [5], even in the absence of active infection to uninfected cells [33]. For most of these studies, *in situ* hybridization was used, including DNA and RNAscope and subsequent FACS and microscopy analysis, but multiple issues of specificity and sensitivity have been raised, especially in individuals ART suppressed for a long time with a small viral reservoir pool. However, these studies provided the initial steps to analyze the viral reservoir microenvironment. Subsequent advances using post-mortem or large biopsies show viral reservoirs in different cell types after years of effective ART, including blood, lymph nodes, platelets/megakaryocytes/bone marrow, and immune-privilege tissues such as the brain supporting the large variability in the viral reservoir pool [5, 7, 34], [35], [36], [37. We believe understanding the HIV reservoir, neighboring uninfected cells, and microenvironment could provide the mechanisms of viral silencing, reactivation, and extended survival, as well as bystander damage as described in several tissues including the heart and the brain.

## Detection of viral reservoirs within the brain by multiplex assays

In the human brain, laser capture microdissection (LCD) for different cell types determined the presence of HIV DNA in microglia/macrophages and a small population of astrocytes [38, 39]. Later, new techniques, such as deep sequencing and genetic tools, confirmed these data in both cell types [10, 40, 41]. It is currently well-accepted that microglia/macrophages are the leading viral reservoirs, but astrocytes remain controversial because many groups did not detect infection 42], [43], [44. This manuscript will try to highlight some of the misconceptions about viral reservoir detection in glial cells and why detection of some viral reservoirs are so elusive and unreliable, especially using emerging spatiotemporal technologies such as DNA/RNA scope and Akoya/nanostring platforms.

Our laboratory developed a multi-component imaging-based methodology to identify, quantify, and characterize rare and low replicating HIV reservoirs in patient samples (blood and peripheral tissues) with unprecedented accuracy and reliability compared to DNAscope, RNAscope, viral protein staining, and several PCR-related techniques. Also, we can spatially couple our technique to mass spectrometry imaging, electron microscopy and laser capture for subsequent targeted and spatial OMICs. Using these methods in human brains, we detected HIV DNA, viral mRNA, and proteins in a cell-type-dependent manner and with a high spatial resolution, even in tissues obtained from HIV-infected individuals under long-term ART [5]. Together, we concluded with seven findings using our spatial techniques. First, we develop a novel multiplex method to detect and quantify viral reservoirs (silent, active, and residual mRNA and protein production) within the CNS of HIV-infected individuals. Second, we provided the distribution of viral reservoirs within the brain. Third, we provided the quantity and cell type with integrated HIV DNA. Fourth, we provided the quantity and cell type with integrated HIV DNA that still produces viral mRNA and proteins within the CNS of individuals under ART. Fifth, we identified that several viral proteins, but not all, are produced, secreted, and taken up by neighboring uninfected cells. Sixth, we identified that efficient systemic ART reduces the brain reservoir pool and prevents the synthesis of some viral proteins, but not all of them. Lastly, our data provided the size of the viral reservoirs within the CNS and the foundation to support the fact that those viral proteins are still secreted in the current ART era [5, 45], [46], [47. Thus, in the long term, it is clear that the failure to eliminate virally infected cells containing intact or defective proviruses induces bystander damage. More importantly, using human brains and lymph node tissues, we learned several “tricks” to reliably detect and quantify “rare” viral reservoirs and overcome several of the issues that need to the considered before starting an experiment.

## The size and technique to detect viral reservoirs within the brain is essential to reach high sensitivity and specificity

Most researchers agree that blood viral reservoirs are not representative of the viral reservoirs pool in different tissues [11, 13]. However, detecting and characterizing viral reservoirs in tissues is challenging for multiple reasons, such as tissue sampling, the distribution of the viral reservoirs, and the low frequency of the cell type analyzed [4, 13]. Experiments in large tissues on non-human primates and resected/post-mortem human samples such as the gut and associated lymph tissue, spleen, urethra, and other lymph tissues indicate that all these are primary tissues containing viral reservoirs, active and latent [5, 38, 48, 49]. In agreement, human autopsy data demonstrated that HIV pro-virus was detected in several tissues with high variability [10].

The brain has been proposed to be a significant viral reservoir due to its immune privilege and the Blood Brain Barrier [50]. They limit immune trafficking/surveillance and poor ART penetration and distribution, resulting in selective viral evolution compared to other “open” tissues to the general circulation, protecting viral reservoirs from the limited immune system presence within the brain. In addition to this, they cause a significant amount of bystander damage to the nervous system, especially in neurons, resulting in 50 % of the people living with HIV having some degree of cognitive impairment [50, 51]. In the brain, multiple groups agreed that microglia/macrophages are the major viral reservoir within the brain [5, 30, 47, 52]. However, whether astrocytes are a viral reservoir and their importance in viral rebound and chronic brain damage is still controversial [38, 47, 53]. In multiple publications, meetings, and other types of communications [42, 43], there is disagreement about the astrocyte’s role as a viral reservoir, which will be discussed further.

Early in the HIV pandemic, laser-captured material and subsequent determination of integrated HIV DNA using Alu-PCR demonstrated that brain microglia/macrophages and a small population of astrocytes harboring HIV DNA [38]. PCR techniques confirmed the presence of integrated HIV DNA in several tissues, including the brain [10, 54]. Only recently has it been demonstrated that HIV-infected astrocytes allow HIV egress into the periphery, showing that although these reservoirs are low in numbers and highly compartmentalized, they can still repopulate the entire body with the virus [53]. Our data demonstrates the essential role of HIV-infected astrocytes in bystander damage of uninfected neurons/glial cells in humans/macaques/rats/humanized mice and several *in vitro* models [55]. Thus, eliminating all viral reservoirs, including those present in the brain, is required to achieve an HIV cure.

**Figure 2:**
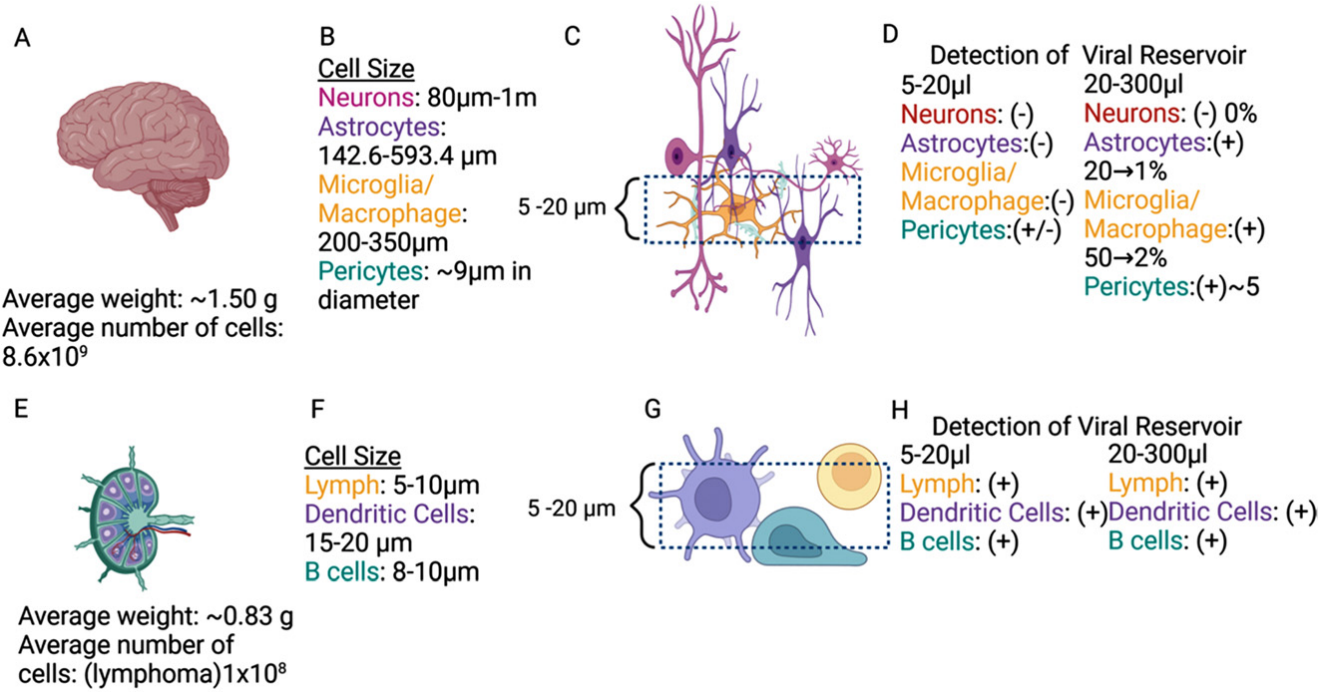
Cellular metrics and viral reservoir detection of brain and lymph node. An overview of the average human brain (A) weight, number of cells, and (B) cell size ordered by type. (C) An overview of a sample and a dashed line section of an observable field of view on a microscope. (D) Breakdown of presence or absence of viral reservoirs ordered by cell type. An overview of average human lymphoma (E) weight, number of cells, and (F) cell size ordered by type. (G) An overview of a sample and a dashed line section of an observable field of view on a microscope. (H) Breakdown of presence or absence of viral reservoirs ordered by cell type (Figure created in Biorender).

Using spatial techniques and tissue 3D reconstruction, our data confirmed that microglia/macrophages and a small population of astrocytes contain HIV-integrated DNA into the host DNA [5, 47]. Our data identify viral reservoirs, classify them into particular cell types, and provide critical information on residual viral protein expression and secretion into neighboring uninfected cells [5, 46, 50, 55, 56]. However, we have to acknowledge that detection is extremely complex due to multiple issues that need consideration before starting the experiments (see below). For example, as represented in Figure 2. The brain had an estimated weight of 1.500 g and 8.6 × 10^9^ cells in average in a healthy adult. Thus, even if the percentage of HIV reservoirs can be low, the total number of infected cells could be higher than any other peripherical organs due to the large total cell numbers, including the example of astrocytes comprising around 5 % of HIV-infected cells [5, 38]. A critical point that is under-considered for most groups working in the area of viral reservoirs, is the varying cell size according to cell type identity and specie analyzed. In the human brain and peripherical nervous system, neurons size ranges from 80 µm to 1 m. Astrocytes are 142.6–593.4 µm. Microglia/macrophages are 200–350 µm, and pericytes average 9 µm in diameter 57], [58], [59], [60], [61], [62], [63], [64], [65], [66. Thus, any tissue detection by RNA/DNA scope-related techniques without properly considering cell size will be highly compromised by the thickness and number of serial sections analyzed. For example, typical tissue sections of 5–20 µm only provide reliable detection of viral reservoirs in small cells such as neurons, macrophage/microglia and T cells. However, it is highly unreliable for the detection of rare viral reservoirs in large cell types such as astrocytes and pericytes (Figure 2C). Thus, proper viral reservoir detection has to consider cell size and abundance or significant 3D reconstitution or large tissue analysis. Our new imaging and multi-detection techniques using 20–300 µm tissue sections and serial sections indicate that neurons are not infected, but astrocytes are infected at a low rate 1–20 %, as well as macrophages/microglia at rate of 2–50 %, and pericytes at a rate of around 5 % even in the current ART era [5, 45, 47, 53]. In agreement with others, if instead, we used tissue sections of 5–20 µm without serial sections or 3D reconstruction our results are always negative in astrocytes and pericytes, supporting the negative data published by several groups. However, as indicated above is just a technical issue not considered for many groups (see Figure 2D). Thus, astrocytes and pericytes are infected at low level. These differences among groups regarding the nature of the brain viral reservoir are mainly due to four factors. First is the critical consideration of the cell type/tissue size. Second, analysis of large pieces of tissues. This is essential to reliably detect rare viral reservoirs or cell populations with low infectivity. Third, serial sections to reconstruct large cell bodies/nuclei/cytoplasms to detect low amounts of HIV-DNA, RNA, and viral proteins. Lastly, the necessity of correlative microscopy to reconstruct cells or areas of tissues with rare viral reservoirs without considering extensive negative areas, especially in tissues from individuals in long term ART.

In contrast, experiments in the gut (with few cell layers) and other tissues with uniform cell sizes, including the lymph nodes, are more straightforward (Figure 2E–H). In lymph nodes, for example, the mean cell size is 5–20 µm for most cell types. Thus, the possibility of detecting all cell types in tissue sections of 5–20 µm is extremely high (Figure 2G). These considerations consistently determine viral reservoirs in 5–20 µm or 20–300 µm tissue sections, including the viral reservoir size and infected cell type (Figure 2H). Thus, cell size, frequency, spatial distribution, and 3D reconstruction are essential for accurately determining the tissue viral reservoir size, especially within the brain.

The following should be considered when using viral reservoirs in animal brain models and comparing them with humans. Human and higher primate astrocytes are different than mice/rats, which should be taken into consideration when using rodent models including humanized mice. The ratio of astrocytes to neurons is higher in primates, and humans have much larger diameters with longer projections overall, amounting to ∼0.4:1 in mice and ∼1:1 in humans [61, 62, 67]. Varicose projection astrocytes, a subtype of astrocytes, are found in higher-order primates and not in mice [68, 69]. They have structurally distinct anatomy, visible with GFAP-positive detection. What makes them unique is their short spiny processes and 1–5 mm long projections in all directions that make contact with the vessels [70]. There is a different organization in the varicose projection subtypes throughout the layers of the cortex between mice and humans [70, 71]. There is an increased number of genes expressed responsible for calcium signaling in the human astrocyte compared to a mouse. Another consideration is the evidence of larger calcium waves in the human astrocytes that are not found in rodents, including calcium waves involved in the establishment of gamma (more than 30 Hz), beta (13–30 Hz), alpha (8–12 Hz), theta (4–8 Hz) and delta (less than 4 Hz). All are associated with critical behaviors in humans, such as concentration, anxiety/attention, passive attention, deep relaxation, and sleep, respectively, denoting the importance of the type and development stage of the cell examined. These differences extend to cell size, frequency, differentiation, and species analyzed [68, 69]. All these significant variations between cell types, species, and animal models are essential for the reliable detection of viral reservoirs (HIV-DNA) but also for residual viral replication (mRNA/proteins).

## The proper determination of the viral reservoir is essential and urgent

Most of the current HIV-associated damage is mediated by ART toxicity or by the recently described bystander damage triggered by viral reservoirs [45]. Our data using the imaging technologies described above indicated that the brain and lymph nodes containing myeloid/glial/lumphoid viral reservoirs that triggers bystander toxicity [46]. Our data indicates that the viral reservoir’s size, distribution, and nature are essential to understanding their microenvironment and associated local inflammation. These spatial-temporal analyses resulted in multiple conclusions. First, viral reservoirs in tissues are diverse; for example, in the brain, we can detect HIV-DNA in microglia/macrophages and a few pericytes and astrocytes. Thus, we have significant cellular heterogeneity, tissue distribution, and bystander damage to uninfected cells, including neurons, astrocytes, and the Blood-Brain Barrier. Second, one-third of viral reservoirs, independent of their nature (full or defective proviruses), had residual viral replication. Thus, viral protein-associated toxicity is real even after years of ART. Third, viral reservoirs use cell-to-cell communication systems to amplify toxicity and metabolic dysfunction into neighboring uninfected cells to a radius of up to 500 µm in human tissues [45].

Thus, damage amplification is highly significant despite the low numbers of remaining viral reservoirs. Lastly, most of these mechanisms are mediated by low expression of viral proteins that compromise cell-to-cell signaling, resulting in synaptic and immune compromise in a highly localized manner. For example, in the controversial area of astrocytes as a brain reservoir, astrocytes form a syncytial network by gap junctions generating the largest cell-to-cell network in most tissues. Our data demonstrated that gap junctions and hemichannels are the main mechanisms to amplify toxicity in uninfected cells [46, 56, 72], [73], [74. HIV-tat protein residual expression maintains Connexin43 expression and channel formation even in inflammatory conditions [5, 46, 75], [76], [77. These large gap junctional networks also denote the need to expand spatial transcriptomics to larger areas to examine these viral reservoir networks within different tissues. These spatiotemporal mechanisms of toxicity and viral reservoir microenvironment need urgent attention to identify mechanisms to reach a cure.

## Conclusions

Despite the significant advances in multiple techniques to detect viral reservoirs, their microenvironment is poorly unknown due to the lack of spatiotemporal techniques to identify and characterize viral reservoirs within tissues. Spatial data already determine that viral reservoirs are different between tissues and within the same tissue. Most of the viral reservoirs had residual viral mRNA and protein expression triggering bystander damage and inflammation into surrounding uninfected cells. Thus, we hope to denote the importance of developing new spatial methodologies to understand the variability of viral reservoirs according to their microenvironment and the opportunities to cure efforts.
